# Reducing systematic review workload through certainty-based screening

**DOI:** 10.1016/j.jbi.2014.06.005

**Published:** 2014-10

**Authors:** Makoto Miwa, James Thomas, Alison O’Mara-Eves, Sophia Ananiadou

**Affiliations:** a The National Centre for Text Mining and School of Computer Science, Manchester Institute of Biotechnology, University of Manchester, 131 Princess Street, Manchester M1 7DN, UK; b Toyota Technological Institute, 2-12-1 Hisakata, Tempaku-ku, Nagoya 468-8511, Japan; c Evidence for Policy and Practice Information and Coordinating (EPPI-)Centre, Social Science Research Unit, Institute of Education, University of London, London, UK

**Keywords:** Systematic reviews, Text mining, Certainty, Active learning

## Abstract

In systematic reviews, the growing number of published studies imposes a significant screening workload on reviewers. Active learning is a promising approach to reduce the workload by automating some of the screening decisions, but it has been evaluated for a limited number of disciplines. The suitability of applying active learning to complex topics in disciplines such as social science has not been studied, and the selection of useful criteria and enhancements to address the data imbalance problem in systematic reviews remains an open problem. We applied active learning with two criteria (certainty and uncertainty) and several enhancements in both clinical medicine and social science (specifically, public health) areas, and compared the results in both. The results show that the certainty criterion is useful for finding relevant documents, and weighting positive instances is promising to overcome the data imbalance problem in both data sets. Latent dirichlet allocation (LDA) is also shown to be promising when little manually-assigned information is available. Active learning is effective in complex topics, although its efficiency is limited due to the difficulties in text classification. The most promising criterion and weighting method are the same regardless of the review topic, and unsupervised techniques like LDA have a possibility to boost the performance of active learning without manual annotation.

## Highlights

• Active learning is promising in the areas with complex topics in systematic reviews.
• Certainty criteria is promising to accelerate screening regardless of the topic.
• Certainty criteria performs as well as uncertainty criteria in classification.
• Weighting positive instances is promising to overcome the data imbalance.
• Unsupervised methods enhance the classification performance.

## Graphical abstract

**Figure:**
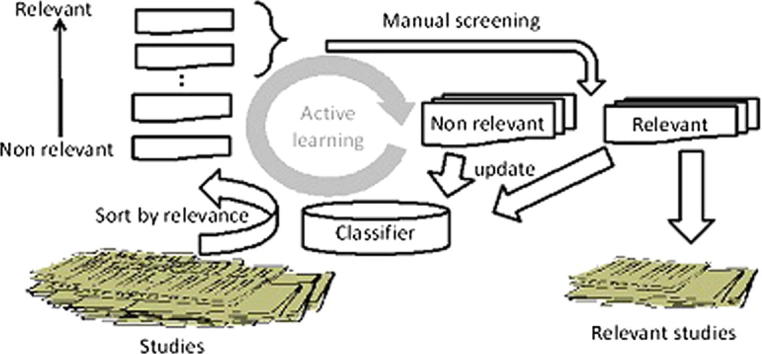

## 1 Introduction

Systematic reviews are a widely used method to bring together the findings from multiple studies in a reliable way and are often used to inform policy and practice (such as guideline development). A critical feature of a systematic review is the application of the scientific method to uncover and minimise bias and error in the selection and treatment of studies [1], [2].

As a result, reviewers make efforts to identify all relevant research for inclusion in systematic reviews. However, the large and growing number of published studies, and their increasing rate of publication, makes the task of identifying relevant studies in an unbiased way both complex and time consuming. Moreover, the specificity of sensitive electronic searches of bibliographic databases is low. In a process known as screening, reviewers often need to look manually through many thousands of irrelevant titles and abstracts in order to identify the much smaller number of relevant ones [3]. Reviews that address complex health issues or that deal with a range of interventions are often those that have the most challenging numbers of items to screen. Given that an experienced reviewer can take between 30 s and several minutes to evaluate a citation [4], the work involved in screening 10,000 citations is considerable (and the screening burden in some reviews is considerably higher than this).

Text mining facilitates the reduction in workload in conducting systematic reviews in a range of areas [5], [6], [7]. Text mining is used increasingly to support knowledge discovery, hypothesis generation [8] and to manage the mass of literature. Its primary goal is to extract new information such as relations hidden in text between named entities and to enable users to systematically and efficiently discover, collect, interpret and curate knowledge required for research [9]. The technology most often tested in relation to the reduction in screening burden is automatic classification, where a machine ‘learns’, based on manual screening, how to apply inclusion and exclusion criteria [10]; that is, it semi-automates the screening process. Pertinent to the focus of this paper, there have been a range of evaluations of the performance of various text mining tools to reducing screening burden, some of which have achieved reductions in workload of between 50% [4] and 90–95% [11], [12] (though others have had rather less success [13]).

The nature of the contribution that such methods can make to systematic reviews is the subject of ongoing debate and evaluation. In some contexts, every citation needs to be screened by two reviewers, and in such situations the workload reduction applies only to the “second” reviewer, with all citations being screened by a human: the theory being that this will maximise recall [14]. In other contexts, citations are checked by a single reviewer, and the theory behind semi-automation is that some of these citations need not be screened manually; here, acceptable recall values are high, in the 95–99% range, but do not necessarily require 100% recall [15]. In a third context, automation is used simply to prioritise workload and ensure that the most likely relevant citations are screened earlier on in the process than would otherwise be the case [12]. Whichever situation pertains, there is a need to optimise the performance of the (semi-) automation methods used in order to maximise both recall and precision (see [13]).

While some studies have yielded impressive results, we lack instances in diverse contexts. In particular, most previous work has been undertaken in systematic reviews of clinical interventions, and the literature in this area is likely to have distinct advantages for machine learning which might not apply universally. Firstly, the use of technical terminology is widespread, and specific terms (e.g., drug names, proteins, etc.) are used in precise ways in distinct literature, in contrast to some disciplines where complex and compound concepts may be used (e.g., ‘healthy eating’ can be described in many ways). Secondly, the medical literature is well indexed on major databases (notably MEDLINE), with the availability of manually assigned Medical Subject Heading (MeSH) terms affording additional information to a classifier; such information is not present on the citations downloaded from other databases. There is therefore a need to assess the performance of text mining for screening in systematic reviews of complex, non-clinical contexts where the use of controlled vocabularies is variable or non-existent.

**Fig. 1:**
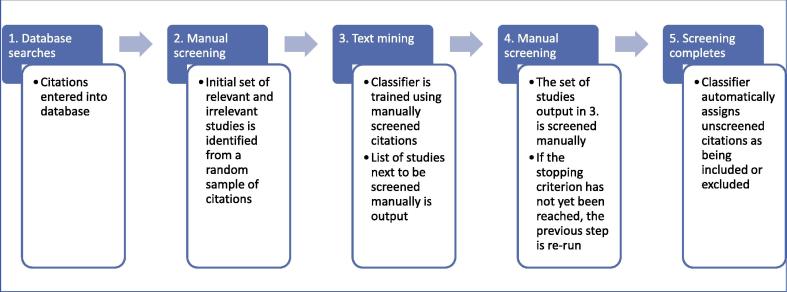
The active learning process.

One of the main strategies adopted in previous work with automatic classifiers is active learning [4]. This ‘supervised’ machine learning technique involves beginning with a small training set and, through iteration, the training set is increased in size and utility (see Fig. 1). Once a given stopping criterion is reached (for example, when all relevant studies have been identified, or when the reviewers have run out of time for manual screening), the process ceases, and the remainder of studies not yet screened manually is discarded. There is thus a good ‘fit’ between the screening process in a systematic review, and the method of active learning. As manual screening progresses, the quantity of training material increases, and there is the opportunity for the classifier to ‘suggest’ items for manual screening, thus making the process more efficient. Although there is an accepted risk when automation is used that some relevant studies may be missed, the gains in terms of reducing burden might make this approach worthwhile. An evaluation of the trade-off between potentially missing studies and reducing burden is required. Given the concerns raised about using such technologies in complex topics, it is important to evaluate performance over a range of conditions.

The primary aim of this study is to assess the suitability of active learning applications to screening in systematic reviews of complex topics, with an emphasis on determining optimal conditions for running these technologies. This paper therefore addresses the following research questions:

1. Does active learning demonstrate similar performance (reduction of burden) in systematic reviews of public health (complex topics) as observed in clinical areas?
2. What features of the active learner improve performance? Specifically,
  (a) does the criterion used to determine the next instances to be annotated in the active learning cycle (i.e., certainty or uncertainty) affect performance? And
  (b) do different types of enhancements to the classifier affect performance?

## 2 Methods

Active learning methods can be classified into two categories from the perspective of data processing: pool-based and stream-based [16]. Pool-based active learning methods assume an unlabelled pool of instances, and determine the most appropriate instances to be annotated from a given data set by sorting them in terms of their informativeness. They often require considerable computational cost and memory. In contrast, stream-based active learning methods receive instances one at a time and decide whether or not the instance should be annotated. However, experiments suggest that stream-based approaches can have poor learner rates and raise too many unnecessary queries compared to pool-based approaches [17].

In this paper, we focus on pool-based active learning methods, since we are interested in learning from specific data sets, in which the sparse positive instances should be identified and presented for annotation as early as possible during the annotation process.

### 2.1 Task description

In the current study, we use active learning for the purposes of two-class text classification tasks, i.e., determining whether a given document is relevant, or irrelevant, in the systematic review for which it is being screened. We have three presumptions about these tasks. Firstly, each data set is imbalanced, and the number of positive instances is much smaller than the number of negative instances. Secondly, each data set contains several separate views of the documents to be classified (e.g., title and abstract and, sometimes, keywords and MeSH terms in our case). Each title or abstract is represented with bag-of-words (BOW). BOW is a simple representation of a text as a set of words with their frequencies in the text. BOW ignores their order and may miss information in the text, but it is often used in text classification. Thirdly, each data set is fixed and small enough for pool-based methods to be applied.

### 2.2 Active learning criteria

We use a support vector machine (SVM) classifier to carry out classification of instances [18]. SVM is one of the most frequently used classifiers. It finds a hyper-plane that separates positive and negative training instances by a maximum margin boundary. We have used two different criteria to determine the next instances to be annotated (i.e., the next title and abstract to be screened manually) in the active learning cycle: **Certainty** and **Uncertainty**, both of which are compared with a baseline **Random** criterion. **Certainty** selects the next instances to be annotated by selecting those instances with the highest probability of being relevant to the review, based on the output of classifiers trained on previously annotated instances. This criterion aims to ensure that positive instances are presented for annotation as early as possible, and thus, this criterion is suitable for the purpose of reducing the burden. This criterion is also considered to be effective for finding good classification models on the imbalanced data since the instances effective for the classification should be close to the small number of positive examples. This **Certainty** criterion has been shown to be an effective method for carrying out active learning on imbalanced data sets, as demonstrated in [19]. This criterion, however, has a potential drawback in that it may produce a hastily generalised classifier that is biased to a limited set of positive instances and misses other positive instances. **Uncertainty**, in contrast, selects the next instances to be annotated by finding those instances that are closest to the separating hyper-plane of the SVM classifier, i.e., the instances for which the classifier is most uncertain about whether they represent positive or negative instances. The presentation of such uncertain instances to be annotated aims to improve the ability of the classifier to find the best separating hyper-plane, and thus to improve its accuracy in classifying new instances. (Some uncertain instances may require more careful consideration in manual annotation and thus more annotation (screening) costs than other instances, as they are close to the boundary of being relevant or irrelevant, but we assume that all of the instances require the same annotation costs.) As a weak baseline comparison of these different instance selection methods, we also employ a method that randomly selects the next instance to be classified (**Random**), and this corresponds to manual screening without active learning.

### 2.3 Pool-based active learning on imbalanced data

We have also investigated the effects of four different types of enhancements to the classifier to improve the efficiency of our active learning task:

1. alleviation of the *data imbalance* problem (i.e., many more negative than positive instances)
2. use of *ensemble classifiers*
3. *covariate shift* method [20]
4. *clustering* the data prior to classification

We explain these enhancements in the rest of this section.

#### 2.3.1 Alleviation of data imbalance problem

We have experimented with two possible solutions to the data imbalance problem. Firstly, a weighting method (Weighting) was employed, which assigns greater weights to positive instances than to negative instances. Each weight was set to the ratio of the number of positive instances to the number of negative instances. Secondly, we performed experiments that employ the aggressive undersampling method [4] (AU). AU trains a classifier using all positive and negative instances, undersamples (i.e., throws away) negative instances that are closest to the separating hyper-plane of the classifier, and re-trains the classifier on the undersampled instances. AU has been proposed to alleviate the problem of simple undersampling in that simple undersampling tends to push the separating hyperplane close to positive instances. We note this method has the potential drawback that it cannot produce a reasonable classifier since the method does not use the instances close to the separating hyper-plane. These are compared with a No-weighting method, which assigns the same weight to all of the instances.

#### 2.3.2 Use of ensemble classifiers

Since the data sets contain separate views of the data to be classified, we are able to build several different classifiers, and to combine them into ensemble classifiers. We employed a simple method to see whether the use of different views affects the performance. We train classifiers on each view individually and select the next set of instances to be annotated based on information output by merging the predictions from all of the classifiers, through the multiplication of predicted probabilities (Voting). In order to evaluate the performance of the Voting method, we also performed experiments using Patient Active Learning (PAL) [4] that randomly selects a classifier to determine the next set of instances to be annotated, instead of using the output of all classifiers to make the decision. PAL aims to avoid hasty generalisation (i.e., the potential for missing clusters of documents that have very different terms from those documents in the initial training set) for a limited set of positive instances by exploiting instances from different views (classifiers).

#### 2.3.3 Covariate shift method

Although the criteria employed in this paper have previously also been employed in active learning, a potential problem is a selection bias problem that the distribution of instances selected through active learning, according to the application of the instance selection criteria described above, may be very different from the overall distribution of instances in the data pool. In order to reduce the potentially negative effect that this bias could have on the performance of the classifier, we have experimented with the use of a simple *covariate shift* method. We use the two-stage approximation method described in [20] (CS). This method tries to match the distribution of the training data to the distribution of the target data that the model is applied to. The method solves an additional two-class classification problem, using a logistic regression classifier, that treats the training data as one class and target data as the other class. A ratio indicating the likelihood that each training instance will appear in the target data is calculated, and the ratio is used to weight each training instance to mimic the distribution of the target data. We use the instances in the data pool as the target data so that the method can alleviate the differences between the distributions of the selected instances and the instances in the data pool. The data pool contains the selected instances, and this has a potential to cause a problem where the inclusion of instances confuses a classifier in the first stage of the CS method, but the performance (in terms of the evaluation criteria explained later) was similar when we excluded the selected instances in our preliminary experiments.

#### 2.3.4 Clustering the data prior to classification

Since we are interested in a specific data pool, we can apply (unsupervised) clustering methods to the data pool in advance. We can obtain cluster-based feature representations that are induced from the entire data pool. The feature representations will differ from the original representation BOW, which has often been employed in active learning for text classification (e.g., [4]), and they will provide different views to avoid hasty generalisation that may miss some positive instances. We incorporate latent dirichlet allocation (LDA) [21] for this purpose. LDA is a Bayesian generative model that represents documents as a mixture of hidden topic distributions, each of which is represented by a floating value. We use the values for topics as another feature representation of documents.

### 2.4 Evaluation settings

#### 2.4.1 Evaluation method

**Fig. 2:**
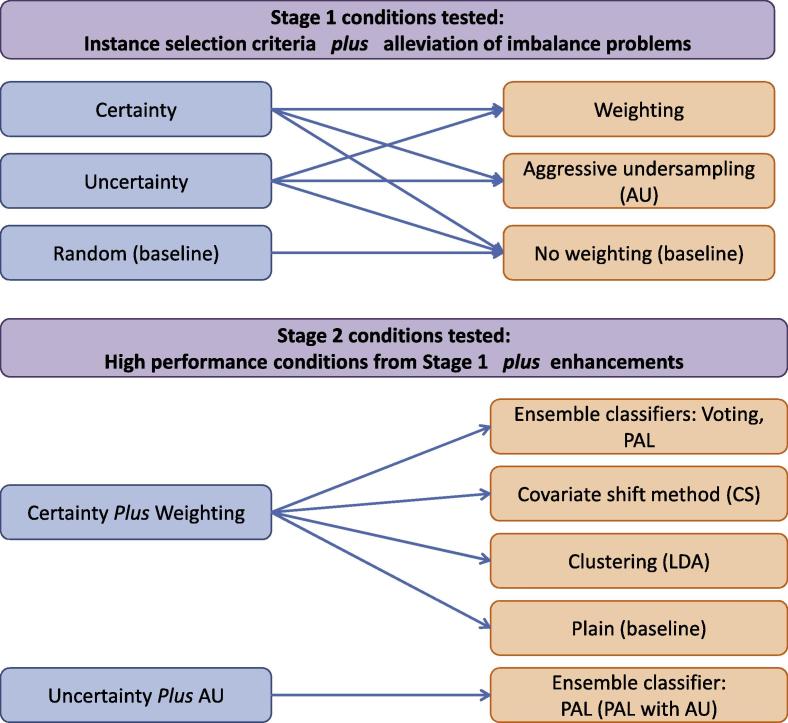
Stages and conditions evaluated.

To evaluate the criteria in Section 2.2 and enhancements in Section 2.3, the evaluation was conducted in two stages. In Stage 1, the different instance selection criteria (**Certainty**, **Uncertainty**) was tested in conjunction with each of the approaches to alleviate the problem of imbalanced data sets (Weighting, Aggressive Undersampling, and No-weighting). In addition, the baseline criterion **Random** was tested in conjunction with the No-weighting method as a baseline. The combination of features from Stage 1 that had the best performance were then combined with other enhancements in Stage 2. The enhancements tested in Stage 2 were the ensemble classification methods (Voting and PAL); the covariate shift method (CS); clustering (LDA); and a no-enhancement baseline (Plain). PAL with AU with the **Uncertainty** criterion was also tested since it was demonstrated as one of the best performing methods on the data sets used [4]. A summary of this process can be seen in Fig. 2.

#### 2.4.2 Pseudo active learning

We have evaluated the active learning methods introduced above within a pseudo active learning scenario. In order to simulate active learning, we repeated the following cycle. Firstly, we applied classifiers to unlabelled instances and selected the *n* top ranking instances according to the instance selection criteria employed in the experiment. We then retrieved and added the correct labels for the selected instances (since, in our pseudo active learning scenario, manually labelled data already exists for the data sets used). Finally, we retrained the classifiers, taking into account the newly labelled instances.

Our experiments selected 5 instances (n=5) per cycle, following [4]. We also tested 1, 5 and 10 for *n*, but our preliminary experiments showed that there was little difference in performance (in terms of the evaluation criteria explained later) for these small *n*. The retraining of the classifiers and selection of instances were performed in less than one second in our preliminary experiments, meaning that the same settings can be used in a real active learning scenario.

#### 2.4.3 Data sets

Our evaluation was carried out using two different data sets.

The first data set (the *clinical data set*) contains three corpora: *proton beam*, *micro nutrients*, and *copd* [4]. This has been used in previous active learning experiments. The second data set, compiled by ourselves (the *social science* data set) contains four corpora (reviews) on public health topics: *Cooking Skills*, *Sanitation*, *Tobacco Packaging*, and *Youth Development*.

Each of the corpora represents each training instance, i.e., a document, as three or four separate views (feature vectors) that correspond to features extracted from titles, abstracts, title concepts (*clinical data set* only) and keywords (optional for the *clinical data set* only). Our experiments make use of the feature vectors in a BOW representation provided in the data sets, without modification. Each instance also has labels annotated during the manual screening processes indicating whether the document is potentially relevant, or irrelevant for the review in question. The screening was carried out by checking only the titles and abstracts of each document to be classified and manually applying the label. In the *clinical data set*, we have labels which indicate whether or not a document was included based on a check of the full text of the paper (a second phase of manual screening).

We use only the labels assigned during the first screening process in our evaluation unless otherwise stated. This enables us to compare the results between the two data sets. Table 1, Table 2 summarise the characteristics of the two data sets.

**Table 1:** The characteristics of the *Clinical data sets*.

	Proton beam	Micro nutrients	Copd
#Positives	243	258	196
#Negatives	4,508	3,752	1,410
Title	✓	✓	✓
Abstract	✓	✓	✓
Title concepts	✓	✓	✓
Keywords	✓	✓	✓

**Table 2:** The characteristics of the *Social science data sets*.

	Cooking Skills	Sanitation	Tobacco Packaging	Youth Development
#Positives	220	498	149	1537
#Negatives	11,295	4966	3061	14,007
Title	✓	✓	✓	✓
Abstract	✓	✓	✓	✓
Title concepts	x	x	x	x
Keywords	x	x	x	x

#### 2.4.4 Settings for machine learning methods

We used the LIBLINEAR library [22] to create the classifiers. We used a dual L2-regularised L2-loss support vector classification solver for SVM and a L2-regularised logistic regression solver for logistic regression. We added bias terms and kept other parameters to the default values.

We used the LDA implemented in gensim [23]. We used all the raw documents in the data sets to construct the LDA model. We removed stop words from the raw documents in a pre-processing step. We then set the number of topics to 300, and performed 20 iterations to obtain the topic distributions.

#### 2.4.5 Performance evaluation measures

We have evaluated the performance of each method on each of the two data sets using three different measures, i.e., *Utility* [24], *Coverage*, and *AUC*.

*Utility* is calculated based on *Yield* and *Burden*. *Yield* represents the fraction of positive instances in the data pool that are identified by a given method, and *Burden* represents the fraction of positive instances in the data pool that have to be annotated/reviewed by reviewers. In a fully manual screening exercise, both *Yield* and *Burden* are 1, i.e., all relevant studies are found through examining every citation manually. We calculated these performance statistics based on labels assigned by reviewers for the queried data (labelled data) and labels assigned by classifiers for the remaining data (unlabelled data).

Given true positives (TP), true negatives (TN), false positives (FP) and false negatives (FN) for labelled data (${\mathrm{TP}}^{L}\text{,}\;{\mathrm{TN}}^{L}\text{,}\;{\mathrm{FP}}^{L}\text{,}\;{\mathrm{FN}}^{L}$) and unlabelled data (${\mathrm{TP}}^{U}\text{,}\;{\mathrm{TN}}^{U}\text{,}\;{\mathrm{FP}}^{U}\text{,}\;{\mathrm{FN}}^{U}$) and total number of instances *N*, *Yield* is calculated as

(1) $$\mathrm{Yield}=\frac{{\mathrm{TP}}^{L}+{\mathrm{TP}}^{U}}{{\mathrm{TP}}^{L}+{\mathrm{FN}}^{L}+{\mathrm{TP}}^{U}+{\mathrm{FN}}^{U}}\text{,}$$

and *Burden* is calculated as

(2) $$\mathrm{Burden}=\frac{{\mathrm{TP}}^{L}+{\mathrm{TN}}^{L}+{\mathrm{FP}}^{L}+{\mathrm{TP}}^{U}+{\mathrm{FP}}^{U}}{N}\text{.}$$

Here, ${\mathrm{FN}}^{L}$ and ${\mathrm{FP}}^{L}$ should be zero since there is one label, but we incorporated these into the definition of *Yield* so that we can apply this measure when we used both labels in the evaluation. *Yield* should be high and *Burden* should be low, and *Yield* is more important than *Burden* in systematic reviewing because of the need to include all relevant documents in a review. Given *Yield* and *Burden* and considering their different levels of importance, *Utility* is calculated as a weighted sum of *Yield* and 1-Burden, and it is defined as

(3) $$\mathrm{Utility}=\frac{\beta *\mathrm{Yield}+(1-\mathrm{Burden})}{1+\beta }\text{.}$$

Here, β is a constant that represents the relative importance of *Yield*, in comparison to *Burden*, and it is set to 19, following [24].

As a further evaluation measure, we also calculated *Coverage*, which indicates the ratio of positive instances in the data pool that are annotated during active learning. *Coverage* is defined as

(4) $$\mathrm{Coverage}=\frac{{\mathrm{TP}}^{L}}{{\mathrm{TP}}^{L}+{\mathrm{FN}}^{L}+{\mathrm{TP}}^{U}+{\mathrm{FN}}^{U}}\text{.}$$

*Coverage* is introduced since the reviewers in practice might run out of time during active learning and they may not be able to accomplish the screening used to calculate the *Utility*.

We calculated *Utility* and *Coverage* on the 80% randomly partitioned documents from the entire data set. The remaining 20% of the entire data set were treated as a test set to evaluate *Area Under the ROC Curve* (*AUC*). This measure evaluates the performance of the final classifiers. This measure does not directly relate to the burden for reviewers, and thus is not the main objective of this paper, but this is important in order to estimate the expected performance of the classifiers when applied to newly published documents when the reviews are updated. The remaining were used for calculating *Utility* and *Coverage*.

Evaluation was performed after every five steps of active learning (25 annotations), and the results reported represent the average over 10 separate trials. The separations for trials were consistent among the evaluations for fair comparison.

## 3 Results and discussion

### 3.1 Evaluation on the clinical data set

We applied several active learning methods to the *clinical data set* in our settings, as well as the settings of [4]. For brevity, we only show the results on the *micro nutrients* corpus in the figures for this data set. Please refer to the Supplementary Material for the results on the other corpora.

We firstly performed several experiments using different combinations of those methods that aim to alleviate the data imbalance problem, combined with either the **Uncertainty** or **Certainty** instance selection criterion. As a baseline, we employed the No-weighting method with the baseline criterion **Random** (i.e., Stage 1 of Fig. 2).

**Fig. 3:**
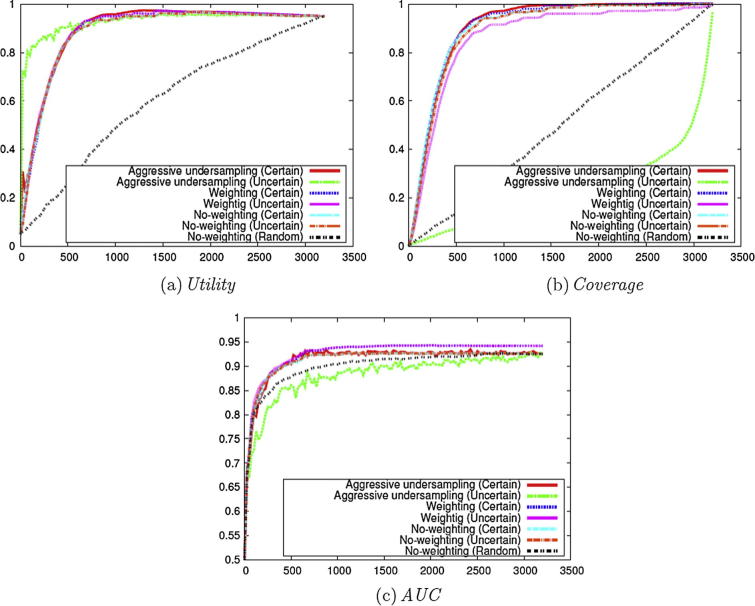
Evaluation on the micro nutrients corpus with different criteria and weighting methods (Stage 1 of Fig. 2).

As shown in Fig. 3(a), all methods perform better than the baseline in terms of *Utility*, thus demonstrating the general desirability of employing active learning. Considering the different active learning configurations, AU with **Uncertainty** shows the highest *Utility* during the early stages of annotation. However, other methods surpass the *Utility* performance of this method after about 20–30% of the data have been annotated. A high *Utility* is always desirable regardless of the number of cycles in active learning, since *Utility* takes into account the annotations after stopping the active learning and the early stage does not necessarily mean a small number of required manual annotations. These results show the superiority of the other methods compared to AU with **Uncertainty**.

The results in Fig. 3(a) also show that the two active learning criteria **Certainty** and **Uncertainty** produce few differences in terms of *Utility*. Furthermore, the employment of the Weighting method has little effect on *Utility*. If this method worked well and it succeeded to adjust the most uncertain probability to be 0.5, the most uncertain instances would be found and the *Utility* would be higher. This small effect on the *Utility* implies that it is difficult to find the most uncertain instances for the imbalanced labels with the Weighting method.

In order to further investigate possible performance differences in the methods alleviating the data imbalance, we calculated *Coverage* during active learning. Fig. 3(b) illustrates the results. These results show that, for methods employing the **Uncertainty** instance selection criterion, the coverage of positive instances is less than perfect, even when most of the instances have been checked (i.e., the *Coverage* value remains at less than 1). This means that there still is a need to check the remaining instances when active learning is terminated. Indeed, AU employing the **Uncertainty** criterion presents an even lower number of positive instances to annotate than the Random method. In contrast to **Uncertainty**, however, the *Coverage*s of **Certainty**-based methods reach 1 after only part of the complete data set has been used (i.e., all positive instances are presented to reviewers for annotation during the active learning process). This means that the remaining instances do not need to be checked even if the classifiers would predict them as positive instances. However, it is problematic to determine the point at which all positive instances have been reviewed, and hence the point at which active learning can stop. This can be the focus of future work.

The results in Fig. 3(b) demonstrate that the calculation of *Coverage* is useful in helping to determine the most appropriate configuration for active learning, since it reveals differences in the utility of *Certainty* and *Uncertainty* that are not taken into account by the *Utility* measure.

We also calculated *AUC* in Fig. 3(c) by applying the classifiers resulting from machine learning to the test set. The results provide an indication of how well the classifiers will perform when applied to newly published documents. These results show that methods employing Weighting always produce the highest *AUC* during active learning, and the *AUC* on the corpora ranges is high and it is close to or more than 0.95. This *AUC* curve also shows that annotating 30% of data is enough to achieve the model that performs as well as the model trained on the annotations of the entire data. Although the high *AUC* is not the main goal of this study, the results show **Certainty** works as well as or better than **Uncertainty** in *AUC*, and **Certainty** is shown to be as useful as **Uncertainty** on imbalanced data sets.

From the analyses in Fig. 3 above, we can conclude that Weighting with **Certainty** constitute the most promising settings for active learning on imbalanced data sets.

We subsequently compared the performance of active learning according to the addition of the other types of enhancements introduced above, i.e., CS, Voting, PAL, and LDA (i.e., Stage 2 of Fig. 2). For comparison purposes, we also show the results without any enhancements (Plain) and the results with one of the best performing methods on the data sets used [4] (PAL with AU with **Uncertainty**). For all of the enhancements mentioned, the reported results employ the Weighting method with the **Certainty** selection criterion, given that this configuration achieved the best overall results, which is consistent with the results illustrated in Fig. 3.

**Fig. 4:**
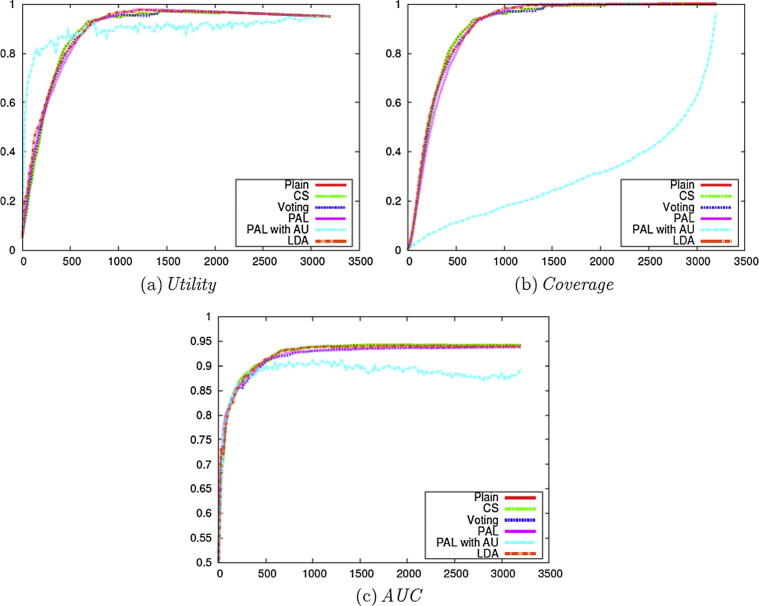
Evaluation on the micro nutrients corpus with different enhancements (Stage 2 of Fig. 2).

Fig. 4 compares the results achieved by the different enhancements. PAL with AU shows a similar tendency to AU with **Uncertainty** as that observed in Fig. 3, in that its performance is worse than other methods. The results obtained by the other methods are broadly comparable to each other. Since all methods except for Plain employ multiple classifiers, our results indicate that Plain can safely be used, in order to achieve maximum efficiency.

#### 3.1.1 Comparison with a previous analysis carried out on the clinical data set

As mentioned above, the *clinical data set* was the focus of a previous, similar series of experiments. We therefore compared the results we achieved with those previously published [4] to ensure that the results are consistent.

The original evaluation differs from ours in two ways. First, it uses the labels assigned during the first screening process only for the purposes of training the classifiers, while the labels assigned during the second screening process are used only for evaluation and are not provided to the classifiers during training. Second, the test set is not used and the entire data are used for calculating *Utility* and *Coverage* in the original evaluation. *AUC* is not used in this evaluation.

**Fig. 5:**
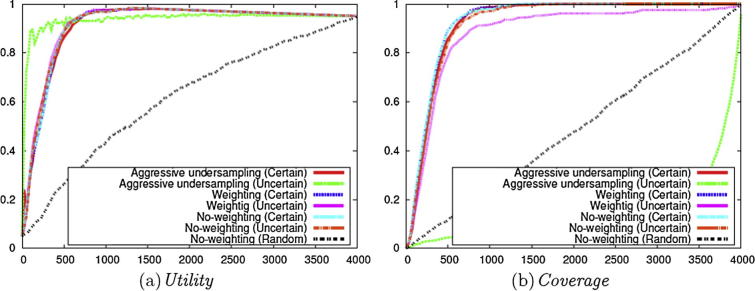
Evaluation of different criteria and weighting methods with a previous analysis carried out on the micro nutrients corpus (Stage 1 of Fig. 2).

**Fig. 6:**
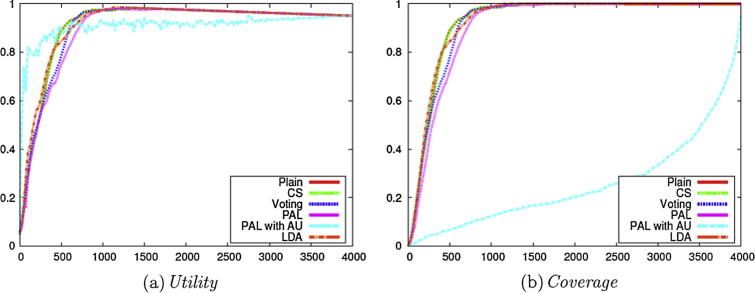
Evaluation of different enhancements with a previous analysis carried out on the micro nutrients corpus (Stage 2 of Fig. 2).

We show the results in Fig. 5, Fig. 6, which correspond to Fig. 3, Fig. 4 respectively in the sense of the stages in Fig. 2. The results are consistent with the corresponding results regardless of the differences in the evaluation settings, except for the *proton beam* corpus.

For the *proton beam* corpus, the AU with **Uncertainty** shows the best *Utility*. The seeming superiority of this method in comparison to others, however, is not necessarily correct. This is because the *proton beam* corpus contains an instance whose two labels from the different screening procedures are inconsistent, i.e., the label from the first screening (of titles and abstracts) shows that the instance is negative, but the label for the second screening (of full-text documents) shows that the instance is positive. Such inconsistencies can degrade the performance of the active learning method. This can not only confuse the classifiers but also degrade the *Yield* since there are only 23 positive instances for the second screening. (It should be acknowledged, however, that such mistakes are likely to occur, since human-applied categories are rarely 100% correct.)

For the other two corpora, these results are consistent in that AU with **Uncertainty** shows the best *Utility* in the early stages of annotation, and other methods surpass the *Utility* after a certain point. This high *Utility* (with the high *Yield*) in the early stages of annotation is consistent with the results reported in [4]. When employing the methods with **Certainty**, *Coverage* reaches 1 after only part of the complete data set has been used. These consistent results lead to the same conclusion as in our context that Weighting with **Certainty** is the most promising setting.

### 3.2 Evaluation on the social science data set

In the same way as for evaluation carried out on the *clinical data set*, we firstly compared the use of the **Uncertainty** and **Certainty** selection criteria, combined with methods aimed at alleviating the data imbalance problem (i.e., Stage 1 of Fig. 2). Similarly to the *clinical data set*, we selected the *Cooking Skills* corpus to show the results in the figures since the same analyses apply to the other corpora. Please refer to the Supplementary Material for the results on the other corpora.

**Fig. 7:**
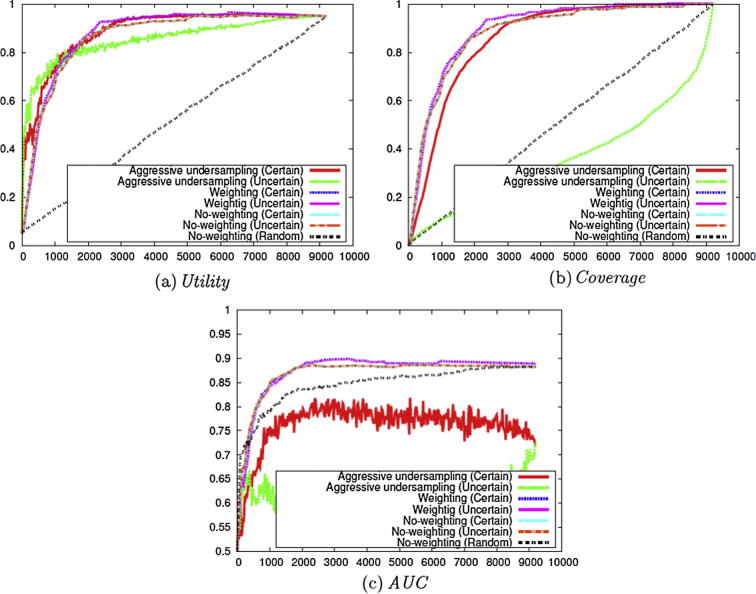
Evaluation on the Cooking Skills corpus with different criteria and weighting methods (Stage 1 of Fig. 2).

Fig. 7(a) compares the *Utility* of these different settings. These results are consistent with the experiments on the *clinical data set*, in that AU with **Uncertainty** shows the best performance in the early stages of annotation, and other methods surpass the performance after a certain point. Compared to the results in Fig. 3(a), the *Utility* is slow to saturate on some corpora.

For the *Coverage* shown in Fig. 7(b), the experiments involving the use of the **Certainty** criterion achieve superior results compared to those using **Uncertainty**. Among the experiments using the **Certainty** criterion, the Weighting method achieves the highest coverage on all the data sets. In contrast to the results shown in Fig. 3(b), *Coverage* reaches 1 only after most of the data sets have been annotated. This shows that the identification of positive instances in the *social science data set* is more complex than for the *clinical data set*.

We have also calculated *AUC*. Fig. 7(c) shows that Weighting achieves the highest *AUC* on most of the data set, regardless of the criteria employed. In contrast, AU performs worse than other methods. A possible explanation is that this method may remove important negative training instances that are close to the separating hyper-plane. *AUC* is less than 0.9 on the data set. This shows that the classification problem is more difficult in the *social science data set* than in the *clinical data set*.

To summarise, the analyses shown in Fig. 7 indicate that the use of the Weighting method, in conjunction with the **Certainty** criterion, appears to be the most promising. This is consistent with the results shown in Fig. 3. Compared to Fig. 3, *Utility* is slow to saturate, *Coverage* is slow to reach 1 and *AUC* is low. These results show the limitation of active learning on the complex data set.

We subsequently compared the other enhancement methods, in the same way as for the experiments carried out on the *clinical data set* (i.e., Stage 2 of Fig. 2). We used Weighting with **Certainty**, with the exception of the method PAL with AU.

**Fig. 8:**
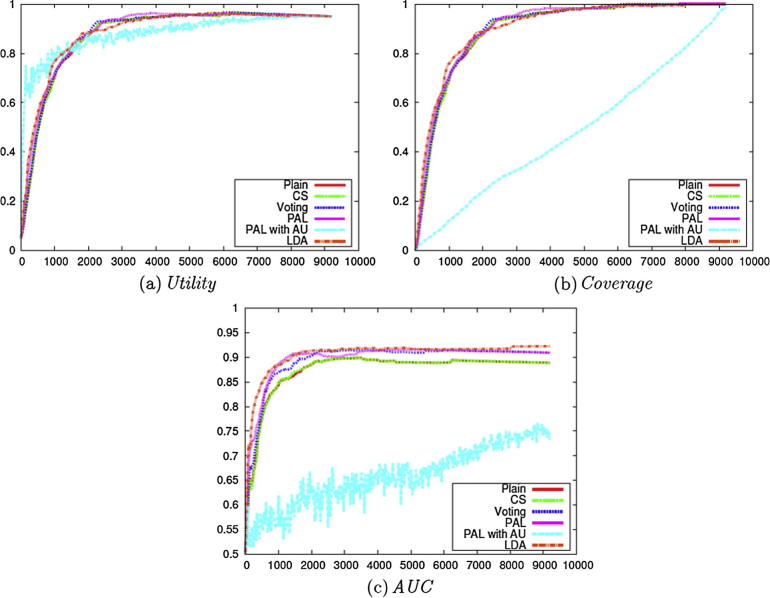
Evaluation on the Cooking Skills corpus with different enhancements (Stage 2 of Fig. 2).

The *Utility* score for the different methods is presented in Fig. 8(a). PAL with AU performs best for the earlier steps of active learning, similarly to AU with **Uncertainty** as in Fig. 4(a), while other methods show similar performance and achieve better *Utility* scores.

In terms of *Coverage*, most methods perform similarly when the **Uncertainty** criterion is employed, as shown in Fig. 8(b).

Although the methods with **Certainty** show similar performance in terms of both *Utility* and *Coverage*, LDA improves the performance for *AUC* as shown in Fig. 8(c). This shows the potential advantage of LDA for active learning tasks, and we can incorporate additional raw data sets, since LDA is unsupervised. Ensemble learning methods like Voting and PAL also show some improvement over Plain especially on the Cooking Skills data set, but the improvement is small compared to that achieved when using LDA. The application of the CS method has no effect on the results, which may be because the **Certainty** and *AUC* are not sensitive to the sampling bias problem. We also investigated ensemble learning methods combined with LDA, but there were few differences in performance.

These results in Fig. 8 compared to Fig. 4 show that most of the methods behave similarly on the two data sets except for LDA. The employment of LDA shows some improvement on the *social science data set* in *AUC*, although it does not affect performance on the *clinical data set*.

### 3.3 Analysis of the difference between two data sets

The results of the application of active learning were broadly consistent for the data sets from the two different areas (clinical and social science), and Weighting with **Certainty** are shown to be the most promising in both data sets.

There were also two differences in the results. First, the results from the two data sets show that the active learning is more difficult on the *social science data set* compared to the *clinical data set*. Second, LDA was shown to be useful in improving the *AUC* on the *social science data set*, but it did not affect the *AUC* on the *clinical data set*.

To investigate possible reasons for these differences, a further experiment was carried out to minimise the differences between the two data sets. Since the *clinical data set* contains additional views based on keywords from MeSH terms and title concepts (MeSH), we checked the effects of these views in the *clinical data set*. Specifically, using Weighting with **Certainty**, we evaluated the performance (1) with or without views from MeSH and (2) with or without a view from LDA.

**Fig. 9:**
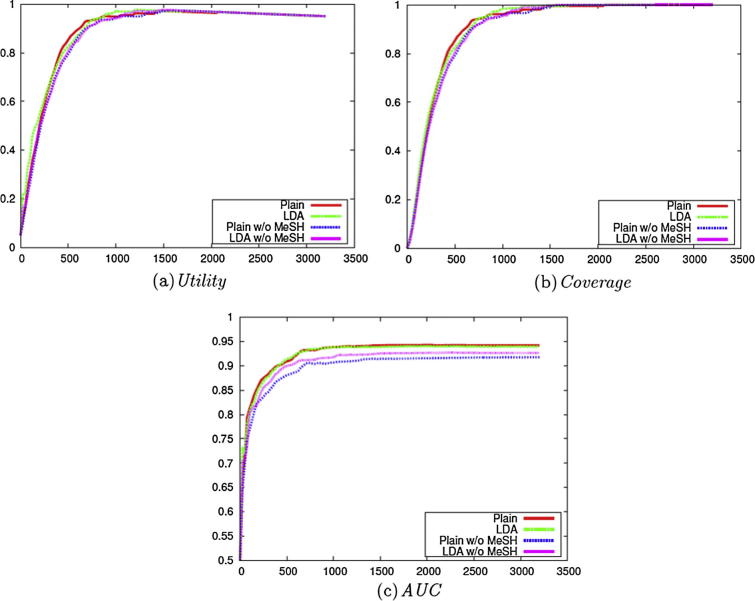
Evaluation on the micro nutrients corpus with different views.

As shown in Fig. 9, the additional views from MeSH have little effect on the performance of the classifier on the *clinical data set*. This limited impact of the additional views indicates that the additional difficulties in active learning in the *social science data set* are caused by the problem itself, i.e., nature of the difficulty in screening in this data set, and not by the additional information available in the clinical data set from the MeSH terms.

The additional views affected the *AUC* on the *micro nutrients* corpus, and LDA also helps in filling the performance gaps between the classifiers with and without MeSH. This indicates that the view from LDA and the views from MeSH are similar to some extent and LDA has the potential to enhance the active learning for the areas where the manually assigned information like MeSH terms are not available.

## 4 Conclusions

This paper addresses the active learning methods on imbalanced data sets in systematic reviews of clinical medicine and social science (specifically, public health) research. The methods are compared using three different performance measures and from three different views. *Utility* and *Coverage* are used to evaluate the reduction of the screening burden, and additionally *AUC* is used to evaluate the classification performance. The Weighting method with the **Certainty** instance selection criterion is the most promising approach for active learning on the imbalanced data sets in both areas and under all evaluation measures, which enables us to achieve our primary goal to reduce the screening burden without hurting the performance of the classifier. *Coverage* and *AUC* especially reveal differences among the methods that are not taken into account in *Utility*. Evaluation using the *Coverage* measure shows that **Certainty** accelerates the identification of relevant studies from the large number of published studies. By employing the *AUC* measure, we show the Weighting method produces a high performing classifier. We also show the potential of LDA to enhance the active learning especially when no manually assigned information such as MeSH terms is available and, hence, to improve the classification performance of active learning in more challenging areas.

Previous evaluations of semi-automating the screening process in systematic reviews have focused on clinical and genetic literature [13]. We have extended the scope of available evaluations to incorporate social science literature since it is important to identify the challenges and provide solutions in non-clinical areas. The novelty of our approach is that, by using identical methods, we have provided a direct comparison between clinical and social science systematic reviews, showing that there are new, but surmountable, problems in the new domain. Our methods demonstrated that active learning is also promising for complex social science topics, frequent in public health systematic reviews, that certainty criteria are useful for both clinical and social data, and that weighting positive instances is useful to overcome data imbalance. We compared seven different corpora, three of clinical data and four of social science data to prove the value of our methods. Despite the complex nature of social science data, our methods have performed well in both settings, and we explained what steps can be taken to overcome the data imbalance problem in systematic reviews. Moreover, we have demonstrated that the differences in the active learning results in these data from the two different areas are not due to the absence of controlled vocabulary terms in the social science data, but to the nature of the different vocabularies used. As this is the first extension of these methods into the social science domain, our focus was on the generalisation of the methods.

As future work, we will undertake further work to achieve greater performance and evaluate the generalizability of our results in other reviews. We will also further investigate the potential of LDA and other unsupervised learning methods. The performance in active learning is affected by the presence of diverse terminology in a data set, which particularly affected the public health data sets, but methods such as LDA have potential to reduce these difficulties. Since LDA is an unsupervised learning method, the addition of more raw documents can improve the model. We can also obtain multiple views by employing different unsupervised models or employing models with different numbers of topics.
