# Supplementary material for: Reducing systematic review workload through certainty-based screening
Source: J Biomed Inform. 2014 Oct;51:242–53. doi: 10.1016/j.jbi.2014.06.005 (PMC4199186; doi:10.1016/j.jbi.2014.06.005)
Supplement: Supplementary file 1 [file mmc1.pdf]

Supplementary material of reducing systematic review workload through certainty-based screening

Makoto Miwa[a,b,*], James Thomas[c], Alison O'Mara-Eves[c], Sophia Ananiadou[a]

[a] *The National Centre for Text Mining and School of Computer Science, Manchester Institute of Biotechnology, University of Manchester, 131 Princess Street, Manchester, M1 7DN, UK*

[b] *Toyota Technological Institute, 2-12-1 Hisakata, Tempaku-ku, Nagoya, 468-8511,Japan*

[c] *Evidence for Policy and Practice Information and Coordinating (EPPI-)Centre, Social Science Research Unit, Institute of Education, University of London, London, UK*

1. Introduction

This supplementary material provides all the results on the corpora in the evaluation criteria in the main manuscript as well as the evaluation criteria **Yield** and **Burden**. Each section corresponds to a figure in the main manuscript, and the sections are ordered by the appearance of their corresponding figures in the main manuscript.

*Corresponding author. Address: Toyota Technological Institute, 2-12-1 Hisakata, Tempaku-ku, Nagoya, 468-8511, Japan, Phone:+81-(0)52-809-1760. This work was carried out while the author was at the University of Manchester, Manchester, UK.

Email addresses: j.thomas@ioe.ac.uk (James Thomas), a.omara-eves@ioe.ac.uk (Alison O'Mara-Eves), Sophia.Ananiadou@manchester.ac.uk (Sophia Ananiadou)

2. Evaluation with different criteria and weighting methods on the clinical data set

This section provides the results on all the corpora corresponding to Figure 3 (i.e., Stage 1 of Figure 2 on the clinical data set).

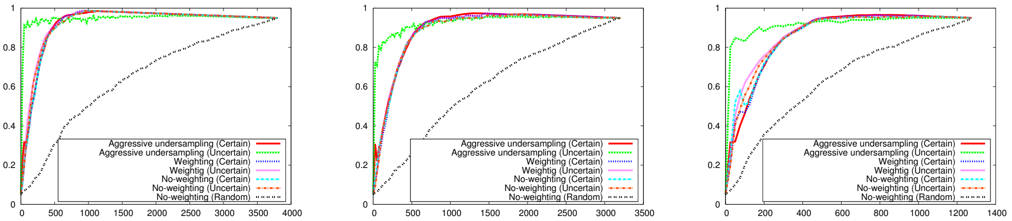

(a) *proton beam* (b) *micro nutrients* (c) *copd*

Figure A.1: *Utility* with different criteria and weighting methods

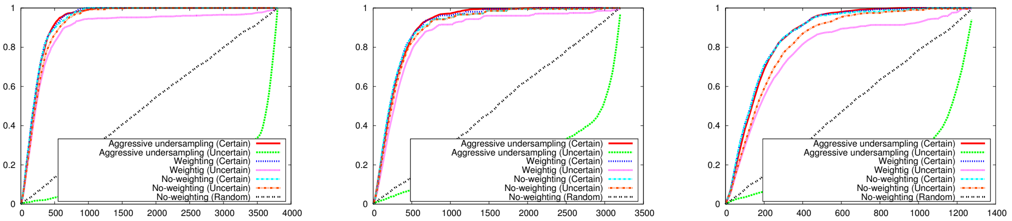

(a) *proton beam* (b) *micro nutrients* (c) *copd*

Figure A.2: *Coverage* with different criteria and weighting methods

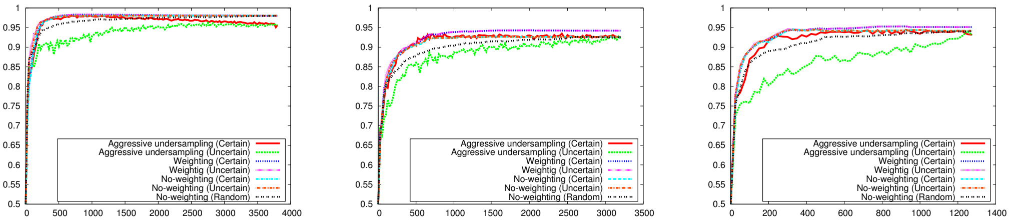

(a) *proton beam* (b) *micro nutrients* (c) *copd*

Figure A.3: *AUC* with different criteria and weighting methods

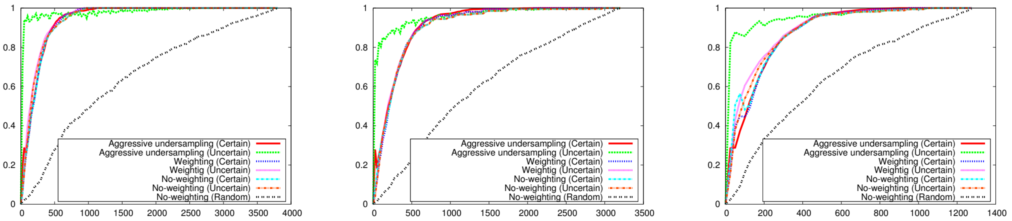

(a) *proton beam* (b) *micro nutrients* (c) *copd*

Figure A.4: *Yield* with different criteria and weighting methods

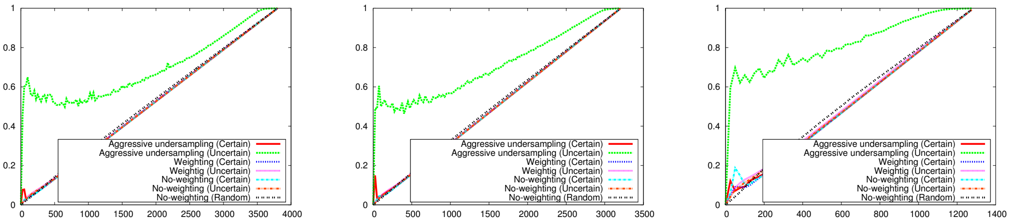

(a) *proton beam* (b) *micro nutrients* (c) *copd*

Figure A.5: *Burden* with different criteria and weighting methods

3. Evaluation with different enhancements on the clinical data set

This section provides the results on all the corpora corresponding to Figure 4 (i.e., Stage 2 of Figure 2 on the clinical data set).

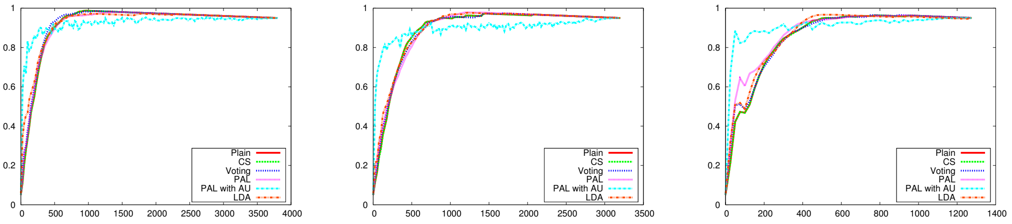

(a) *proton beam* (b) *micro nutrients* (c) *copd*

Figure A.6: *Utility* with different enhancements

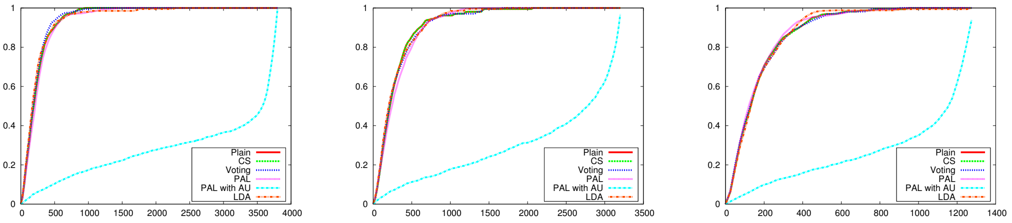

(a) *proton beam* (b) *micro nutrients* (c) *copd*

Figure A.7: *Coverage* with different enhancements

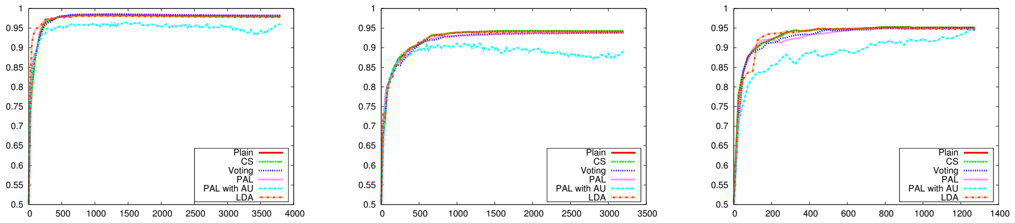

(a) *proton beam* (b) *micro nutrients* (c) *copd*

Figure A.8: *AUC* with different enhancements

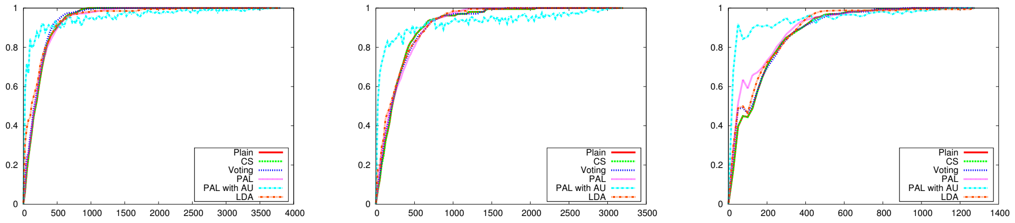

(a) *proton beam* (b) *micro nutrients* (c) *copd*

Figure A.9: *Yield* with different enhancements

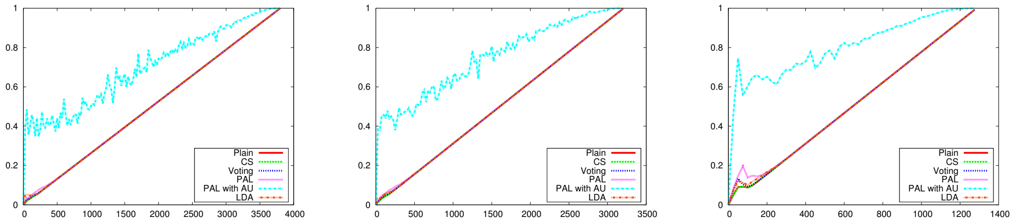

(a) *proton beam* (b) *micro nutrients* (c) *copd*

Figure A.10: *Burden* with different enhancements

4. Evaluation with different criteria and weighting methods using a previous analysis carried out on the clinical data set

This section provides the results on all the corpora corresponding to Figure 5 (i.e., Stage 1 of Figure 2 on the clinical data set using the previous analysis).

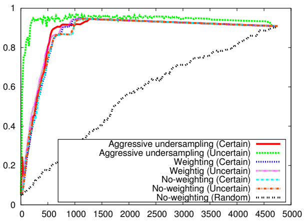

(a) *proton beam*

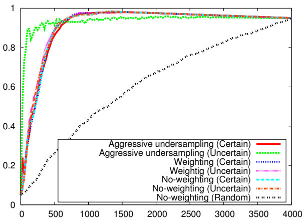

(b) *micro nutrients*

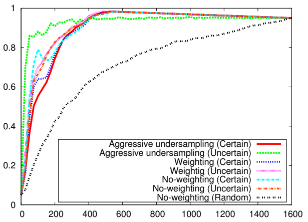

(c) *copd*

Figure A.11: *Utility* with different criteria and weighting methods

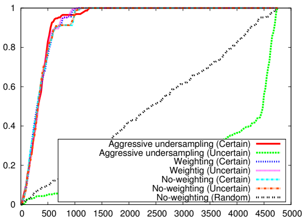

(a) *proton beam*

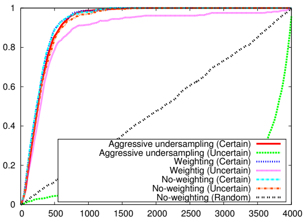

(b) *micro nutrients*

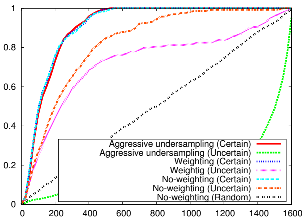

(c) *copd*

Figure A.12: *Coverage* with different criteria and weighting methods

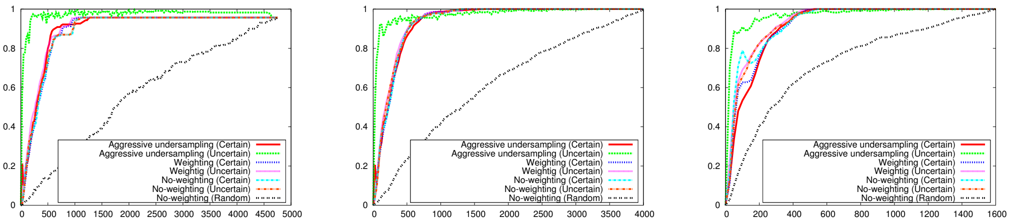

(a) *proton beam* (b) *micro nutrients* (c) *copd*

Figure A.13: *Yield* with different criteria and weighting methods

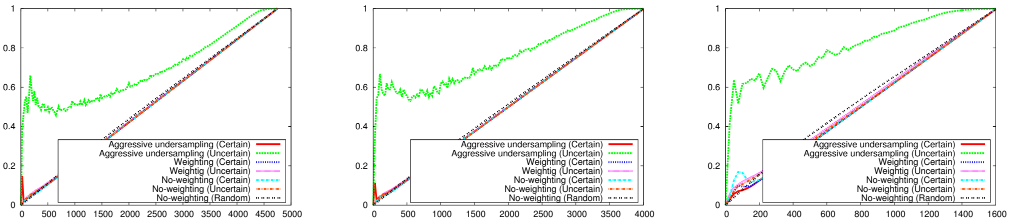

(a) *proton beam* (b) *micro nutrients* (c) *copd*

Figure A.14: *Burden* with different criteria and weighting methods

5. Evaluation of different enhancements with a previous analysis carried out on the clinical data set

This section provides the results on all the corpora corresponding to Figure 6 (i.e., Stage 2 of Figure 2 on the clinical data set using the previous analysis).

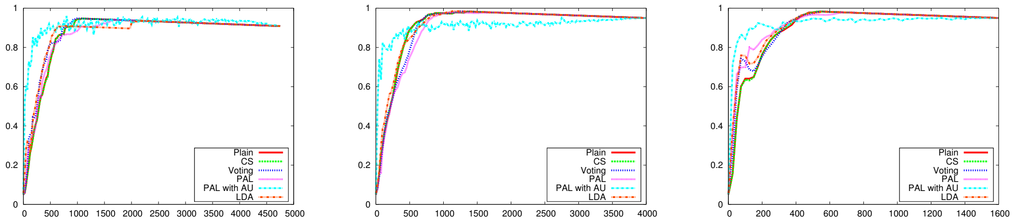

(a) *proton beam* (b) *micro nutrients* (c) *copd*

Figure A.15: *Utility* with different views

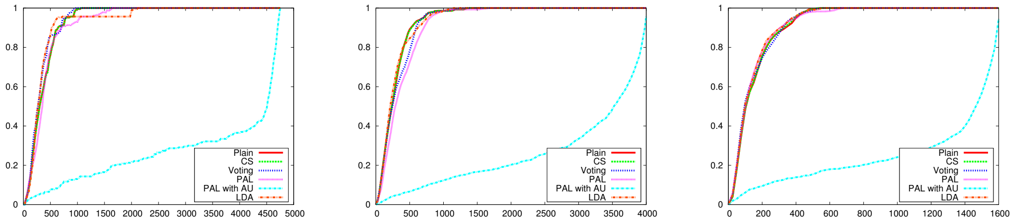

(a) *proton beam* (b) *micro nutrients* (c) *copd*

Figure A.16: *Coverage* with different views

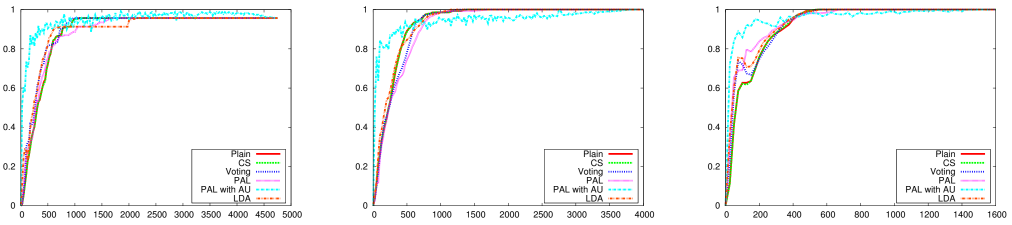

(a) *proton beam* (b) *micro nutrients* (c) *copd*

Figure A.17: *Yield* with different views

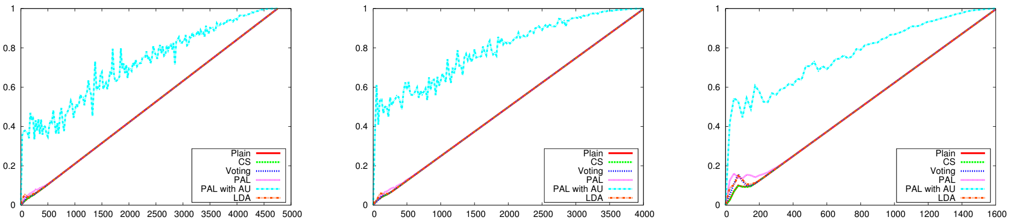

(a) *proton beam* (b) *micro nutrients* (c) *copd*

Figure A.18: *Burden* with different views

6. Evaluation with different criteria and weighting methods on the social science data set

This section provides the results on all the corpora corresponding to Figure 7 (i.e., Stage 1 of Figure 2 on the social science data set).

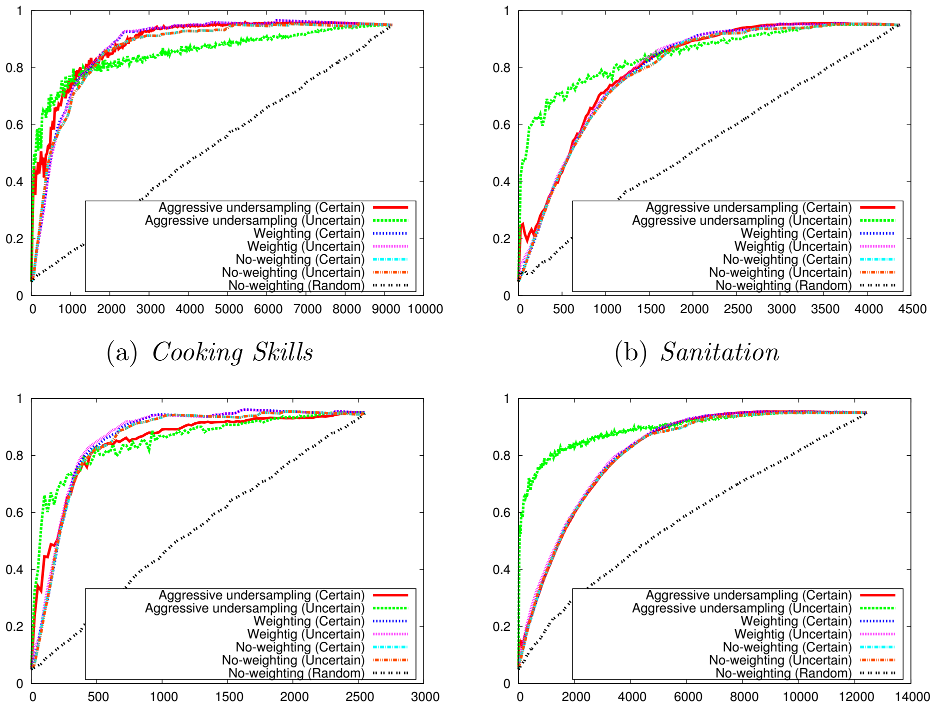

(a) *Cooking Skills*

(b) *Sanitation*

(c) *Tobacco Packaging*

(d) *Youth Development*

Figure A.19: *Utility* with different criteria and weighting methods

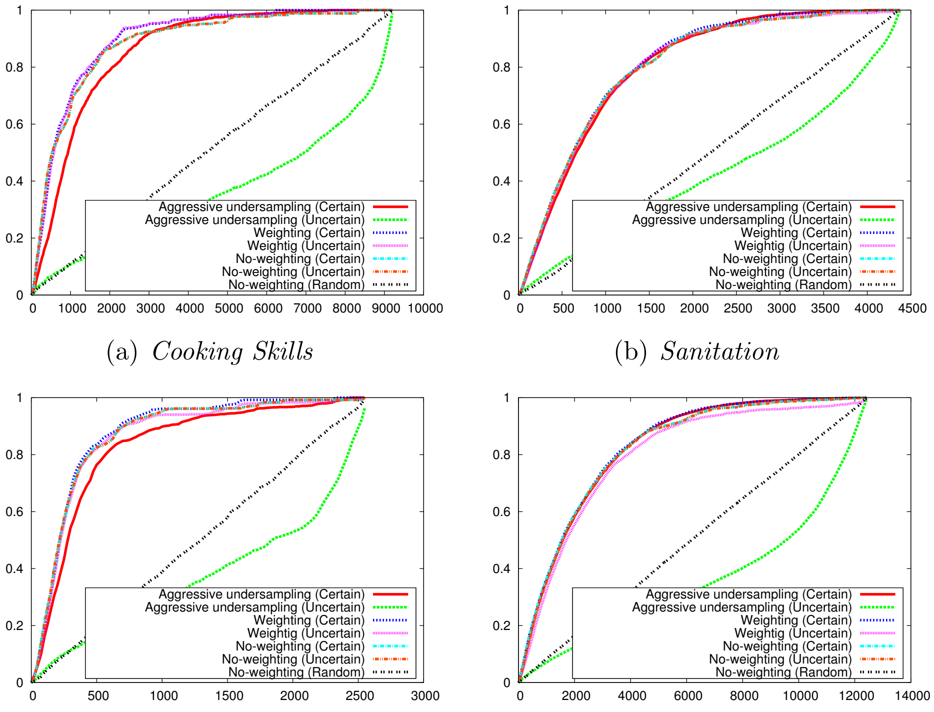

(a) *Cooking Skills*

(b) *Sanitation*

(c) *Tobacco Packaging*

(d) *Youth Development*

Figure A.20: *Coverage* with different criteria and weighting methods

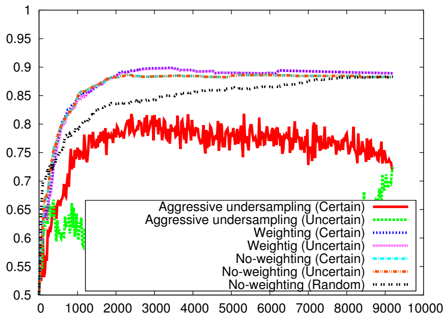

(a) *Cooking Skills*

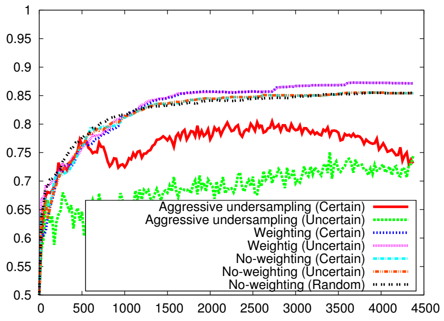

(b) *Sanitation*

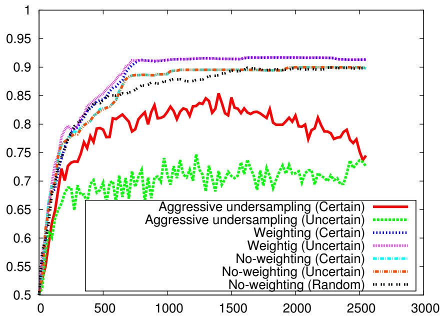

(c) *Tobacco Packaging*

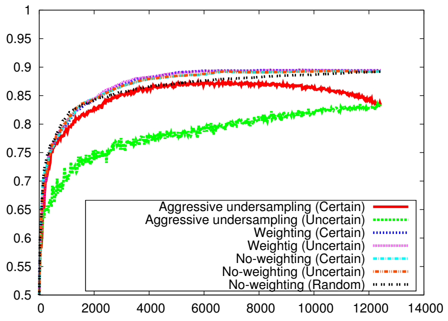

(d) *Youth Development*

Figure A.21: AUC with different criteria and weighting methods

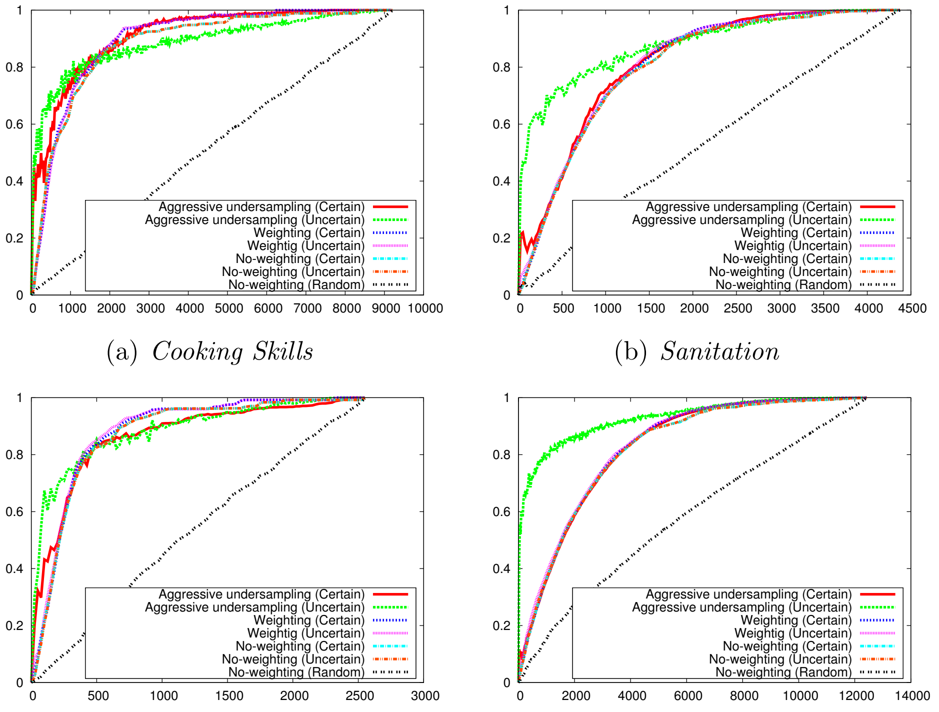

(a) *Cooking Skills* (b) *Sanitation*

(c) *Tobacco Packaging* (d) *Youth Development*

Figure A.22: *Yield* with different criteria and weighting methods

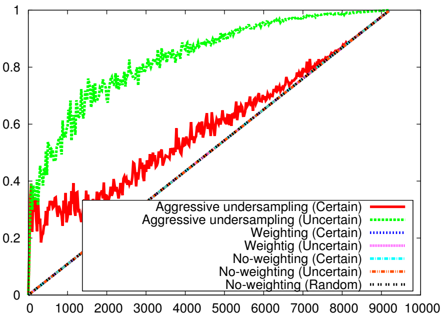

(a) *Cooking Skills*

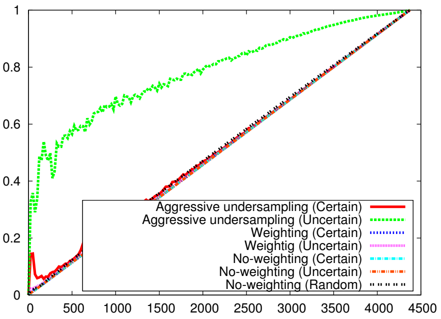

(b) *Sanitation*

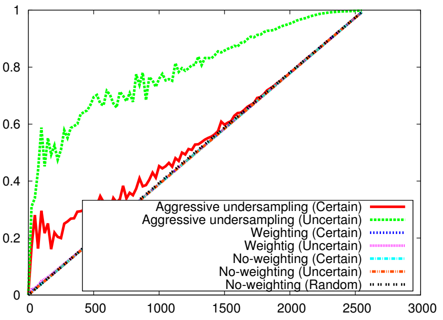

(c) *Tobacco Packaging*

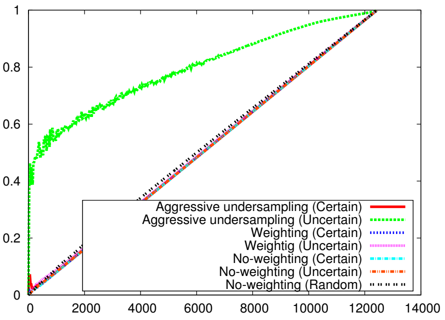

(d) *Youth Development*

Figure A.23: *Burden* with different criteria and weighting methods

7. Evaluation with different enhancements on the social science data set

This section provides the results on all the corpora corresponding to Figure 8 (i.e., Stage 2 of Figure 2 on the social science data set).

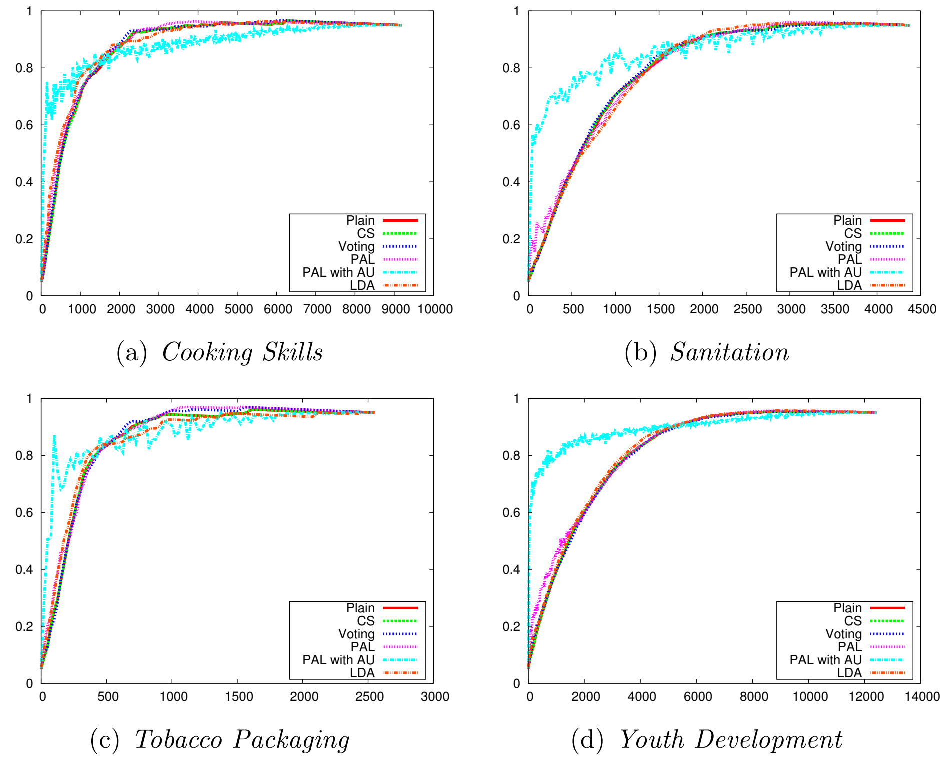

(a) *Cooking Skills* (b) *Sanitation*

(c) *Tobacco Packaging* (d) *Youth Development*

Figure A.24: *Utility* with different enhancements

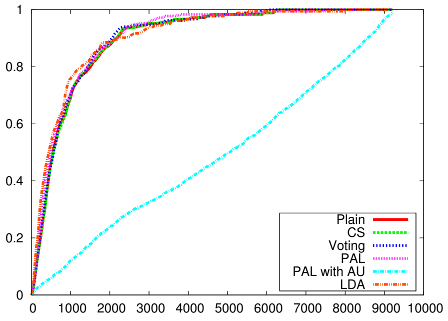

(a) *Cooking Skills*

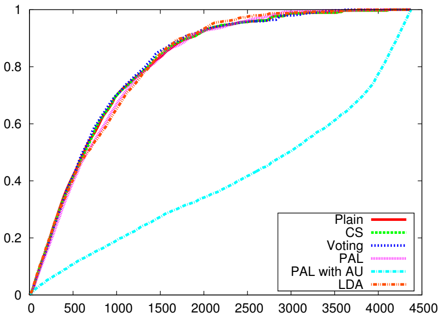

(b) *Sanitation*

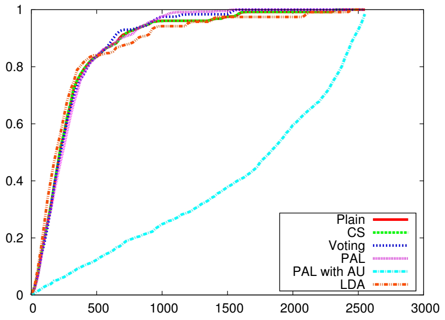

(c) *Tobacco Packaging*

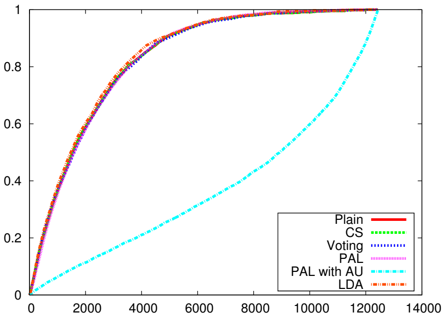

(d) *Youth Development*

Figure A.25: *Coverage* with different enhancements

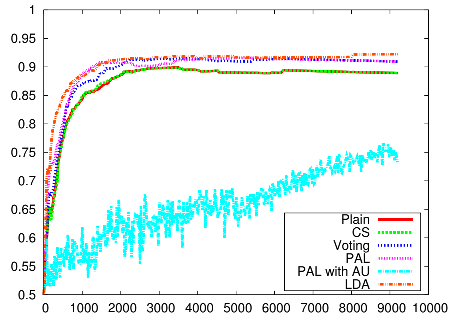

(a) *Cooking Skills*

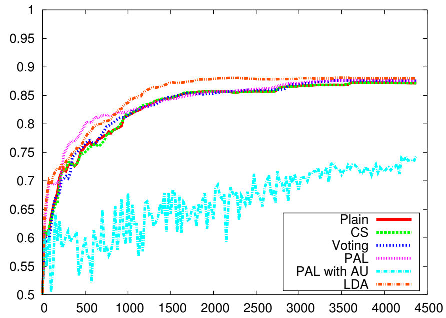

(b) *Sanitation*

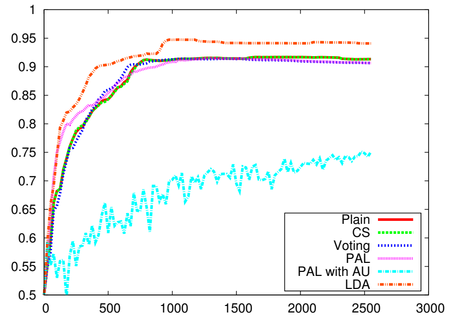

(c) *Tobacco Packaging*

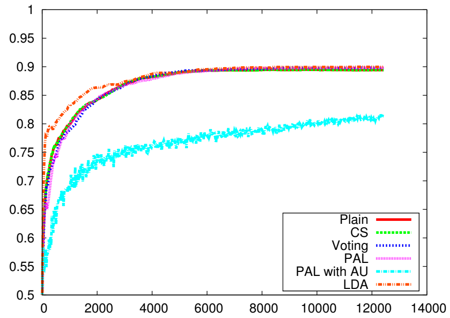

(d) *Youth Development*

Figure A.26: *AUC* with different enhancements

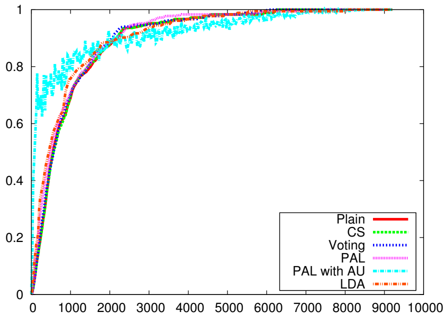

(a) *Cooking Skills*

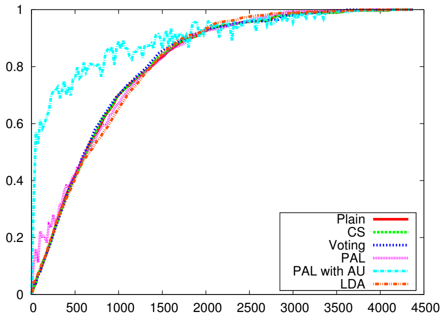

(b) *Sanitation*

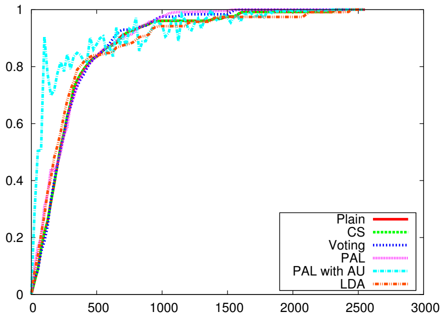

(c) *Tobacco Packaging*

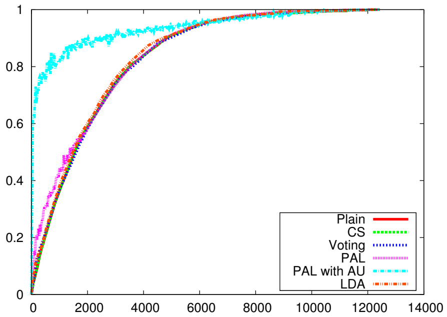

(d) *Youth Development*

Figure A.27: *Yield* with different enhancements

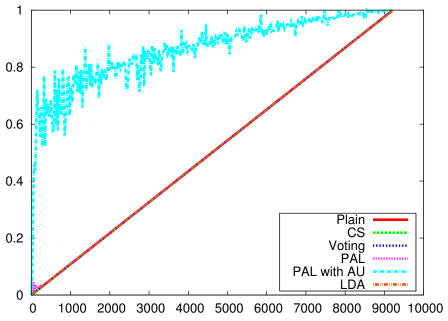

(a) *Cooking Skills*

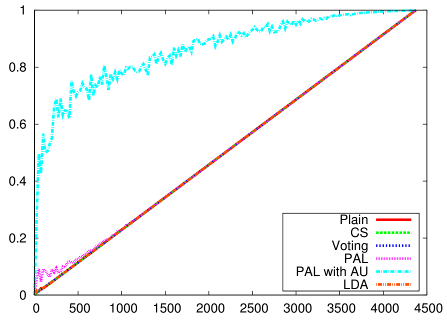

(b) *Sanitation*

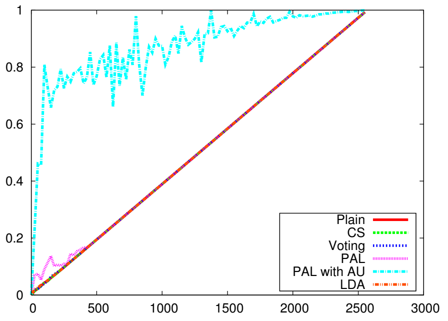

(c) *Tobacco Packaging*

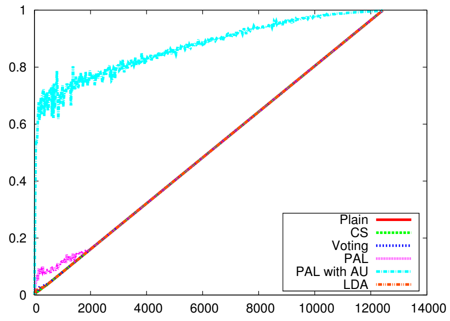

(d) *Youth Development*

Figure A.28: *Burden* with different enhancements

8. Evaluation with different views on the clinical data set

This section provides the results on all the corpora corresponding to Figure 9.

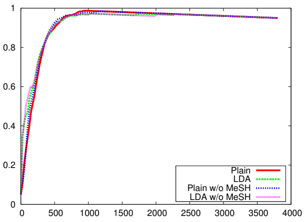

(a) *proton beam*

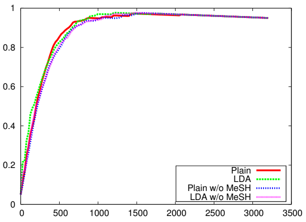

(b) *micro nutrients*

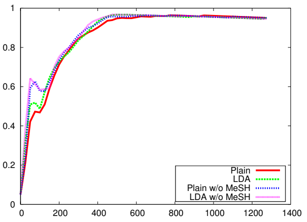

(c) *copd*

Figure A.29: *Utility* with different views

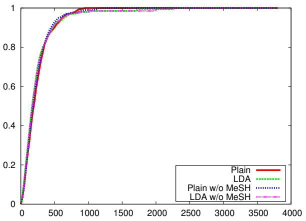

(a) *proton beam*

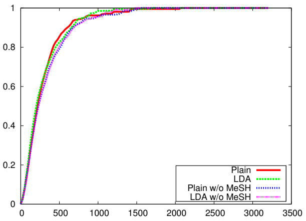

(b) *micro nutrients*

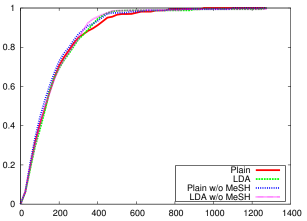

(c) *copd*

Figure A.30: *Coverage* with different views

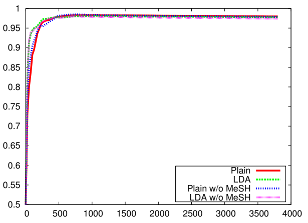

(a) *proton beam*

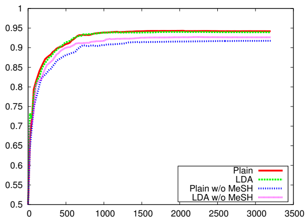

(b) *micro nutrients*

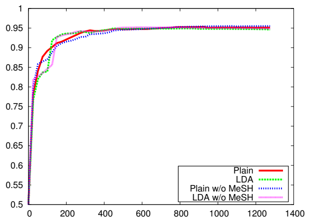

(c) *copd*

Figure A.31: *AUC* with different views

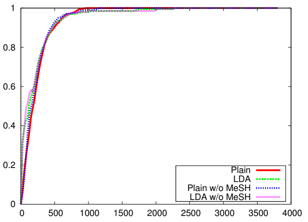

(a) *proton beam*

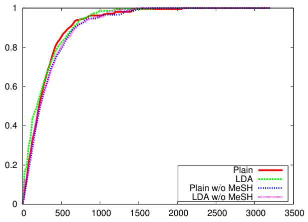

(b) *micro nutrients*

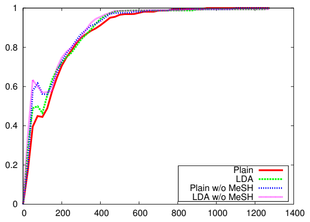

(c) *copd*

Figure A.32: *Yield* with different views

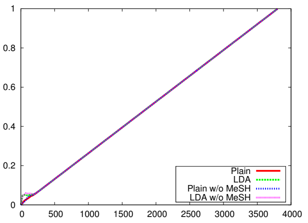

(a) *proton beam*

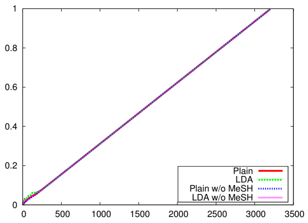

(b) *micro nutrients*

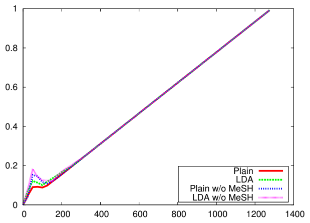

(c) *copd*

Figure A.33: *Burden* with different views
